# Trauma-Related Dissociation Is Linked With Maladaptive Personality Functioning

**DOI:** 10.3389/fpsyt.2018.00206

**Published:** 2018-05-25

**Authors:** Antonella Granieri, Fanny Guglielmucci, Antonino Costanzo, Vincenzo Caretti, Adriano Schimmenti

**Affiliations:** ^1^Department of Psychology, University of Turin, Turin, Italy; ^2^Faculty of Human and Social Sciences, Kore University of Enna, Enna, Italy; ^3^Department of Human Sciences, LUMSA University of Rome, Rome, Italy

**Keywords:** trauma, dissociation, personality, alternative DSM-5 model for personality disorder, defense mechanisms

## Abstract

**Background:** Extensive research has demonstrated the positive associations among the exposure to traumatic experiences, the levels of dissociation, and the severity of psychiatric symptoms in adults. However, it has been hypothesized in clinical literature that an excessive activation of the dissociative processes following multiple traumatic experiences may jeopardize the psychological and behavioral functioning of the individuals, fostering higher levels of maladaptive personality functioning.

**Methods:** The study involved 322 adult volunteers from Italy. Participants completed measures on traumatic experiences, dissociation, and maladaptive personality traits.

**Results:** The number of traumatic experiences reported by participants were positively associated with dissociation scores and maladaptive personality scores. Mediation analyses showed that dissociation acted as a partial mediator in the relationship between traumatic experiences and overall maladaptive personality functioning. Regression curve analyses showed that the positive association between maladaptive personality functioning and dissociation was stronger among participants with higher exposure to traumatic experiences.

**Conclusion:** Exposure to multiple traumatic experiences may increase the risk for an excessive activation of the dissociative processes, which in turn may generate severe impairments in multiple domains of personality functioning.

## Introduction

Dissociation is a mental process already available in early stages of development, which allows an individual to tolerate distressing events by splitting off highly incoherent or overwhelming thoughts, memories, and feelings (1–7). The dissociative process could be conceptualized as a primary response to stress, which protects the mind from disorganization and feelings of fragmentation through a temporary selective exclusion of mental contents from consciousness and through multiple disconnections between mental states (3, 5, 8, 9).

However, when an individual is exposed to multiple traumatic experiences, and especially when such experiences started to occur during childhood—when a child has little capacity for self-regulation—dissociation may become a “psychological organizer” of the entire personality of the individual (5, 9–12). In these circumstances, the excessive activation of dissociative processes jeopardizes the mental and behavioral functioning of the individual so that dissociation becomes pathological and pervasive, and it may lead to severe psychiatric symptoms (5).

This conceptualization of dissociation as a protective function of the mind, which is, however, sensitive to the experiences of chronic traumatization to the point that it can even generate a functional reorganization of personality into distinct structures not fully integrated between them, has been widely endorsed in clinical literature (13–16) and is consistent with contemporary neurobiological findings, supporting the view that trauma is often linked to pathological dissociation (17–22).

Indeed, extensive research has reported a positive association between exposure to different types of trauma (e.g., emotional, physical, sexual) and the severity of dissociation (5, 23–26). Moreover, higher numbers of traumatic experiences in life seem to be associated with an increased risk for psychopathological conditions (5, 27–29).

Notably, exposure to multiple traumatic experiences has also been associated with less flexible, and more maladaptive, personality patterns (30–32), and longitudinal studies have shown a mediating role of personality features in the relationship between traumatic experiences and psychopathology (33, 34).

This study is part of a wider research program aimed at exploring the relationships among trauma, dissociation, and adult psychopathology. In previous research, we have described and empirically tested with positive results a conceptual model in which dissociation plays a mediating role between different types of traumatic experiences and psychopathology (5). A basic tenet of this model is that psychological trauma can be considered as a complex factor, in which different types of distressing experiences have different probability to co-occur and to generate an excessive activation of the dissociative process, with, however, higher numbers of distressing experiences being more likely related to excessive dissociation and psychopathology.

In fact, this model postulates that among the many contextual, social, and psychological variables that are associated with an increased risk for trauma exposure, the probability of experiencing a trauma is also related with the other traumata that occurred in an individual's life. Noteworthy, this particularly applies when trauma exposure starts in childhood. Just to make an example here: a child who loses a parent (trauma n. 1) will have an increased probability of being neglected (trauma n. 2) by the other parent (for example, because the living parent struggles to cope with his or her own depressive feelings, and/or because the death of his or her spouse generated financial problems in the family); being neglected at home, in turn, increases the probability that the child will be exposed to abuses (trauma n. 3) outside the family (e.g., being bullied at school), because he or she lacks a loving and protective figure who can help him or her to adequately cope with difficulties and problems. It is rather clear that this sequence of consecutive exposure to multiple traumatic experiences can continue. However, what is critical here is that the psychological and behavioral functioning of this child will be intensely affected by such negative experiences, to the point that his or her development will be deviated toward atypical trajectories, involving the development of maladaptive personality features.

Therefore, in the present study we hypothesized that the mediating role of dissociation already observed in the relationship between trauma and adult symptomatology (5) can also be observed in the relationship between trauma and maladaptive personality functioning.

## Materials and methods

### Participants

The study involved 322 adult volunteers (111 males, 34.4%; 221 females, 65.6%) from Sicily (Italy). Participants ranged in age from 18 to 64 years old (*M* = 33.48; *SD* = 13.53), and showed an average level of education of 15.45 years (SD = 3.50). One hundred forty-three of them (44.4%) were in a marital relationship, while the others were either single, divorced, or widowed. There were no gender differences in relation to age of participants [*t*_(320)_ = 0.35, *p* = 0.73], their years of education [*t*_(320)_ = 0.69, *p* = 0.49], and their marital status [$\chi_{\left(1\right)}^{2}$ = 1.23, *p* = 0.27].

### Procedures

Participants were recruited in Sicily (Italy) through public and electronic advertisements (flyers in public places and posts in social network pages). People who contacted the research office via e-mail or by phone were asked to participate in a study on personality and its features in relation to life events, and those who expressed their potential willingness to participate (*N* = 407) were sent an anonymous electronic link that allowed them to take part in the study. Inclusion criteria were as follows: (1) age between 18 and 65 years; (2) native or fluent Italian speaker; (3) no current diagnosis of severe psychiatric disorders (such as psychotic disorders, bipolar disorders, dementia); (4) not being in treatment with psychotropic medication within the past 3 months. Three hundred and twenty-two participants met the inclusion criteria and signed the informed consent. They were administered measures for the assessment of traumatic experiences, dissociation, and maladaptive personality. The study obtained ethical permission from the last author's university Internal Review Board for psychological research. Participants did not receive any compensation for their involvement in the study.

### Measures

The Traumatic Experiences Checklist (TEC) (35) is a self-report measure addressing 29 types of potentially traumatic events (e.g., “Loss of a family member when you were a child”). It is used in both clinical practice and research and has been validated in many countries, including Italy, with the Italian translation showing adequate reliability and validity even at the item level (5). Different scores can be calculated on the TEC. Consistent with previous research (36), in this study we used the cumulative score of the TEC as an index of total trauma. The KR-20 index of TEC scores in this study was 0.57.

The Dissociative Experiences Scale–II (DES-II) (37) is a 28-item self-report measure of dissociative experiences (e.g., experiences of absorption, emotional detachment, amnesia, depersonalization, derealization, identity confusion, and compartmentalization). The subject is asked to circle the number to show what percentage of the time he or she experiences these symptoms (e.g., “Some people have the experience of looking in a mirror and not recognizing themselves. Circle the number to show what percentage of the time this happens to you”). The overall score ranges from 0 to 100% and is the average score obtained by adding up the 28 item scores and dividing by 28. International research shows that the psychometric properties of the DES-II are excellent (38), and the Italian translation of the DES-II similarly showed high internal consistency, adequate item-to-scale homogeneity, good split-half reliability, and good convergent validity (3). In this study, Cronbach's alpha of the DES-II was 0.94.

The Personality Inventory for DSM-5-Brief Form-Adult (PID-5-BF) (39) is a 25-item self-report measure assessing five maladaptive personality-trait domains (i.e., negative affectivity, detachment, antagonism, disinhibition, and psychoticism) according to the alternative DSM-5 model for personality disorders (40). Measures range from 0 to 75, with higher scores indicating greater overall personality dysfunction. Each trait domain includes five items and ranges from 0 to 15, with higher scores indicating greater dysfunction in the specific personality-trait domain. The PID-5-BF has been validated in many countries, including Italy (41), with positive results. In the present study, Cronbach's alpha for the PID-5-BF total score was 0.86, whereas Cronbach's alpha for the PID-5-BF domains were 0.63 (Negative affectivity), 0.66 (Detachment), 0.77 (Antagonism), 0.66 (Disinhibition), 0.73 (Psychoticism).

### Statistical analysis

Descriptive statistics were calculated for all the investigated variables. An independent sample *t*-test was used to examine gender differences. Pearson's *r* correlations were used to examine potential associations among traumatic experiences, dissociation, and maladaptive personality domains. Mediation analyses testing whether dissociation mediated between the number of traumatic experiences and maladaptive personality domains were performed using the Process Macro for SPSS (42), applying Model 4 with 5,000 bias-corrected bootstrap samples and controlling for gender, age, years of education, and marital status of participants. Finally, participants' scores on the TEC were split at the 50th percentile to obtain two subgroups with low and high levels of traumatic experiences, respectively. We tested whether regression curves for global maladaptive personality scores as a function of dissociation scores fit differently with respect to these two subgroups.

## Results

Descriptive statistics are presented in Table 1, for the full sample and differentiated by gender, along with levels of significance for gender differences. Males reported higher scores in the PID-5-BF antagonistic domain (*p* = 0.001), whereas females reported higher scores in the PID-5-BF negative affectivity domain (*p* < 0.001). No other gender differences were observed in the sample.

**Table 1:** Descriptive statistics and gender differences.

	**Full sample (*****n*** = **322)**			**Males (*****n*** = **111)**		**Females (*****n*** = **221)**		***t*_(320)_**	***p***
	***M***	***(SD)***	**Observed range**	***M***	***(SD)***	***M***	***(SD)***		
TEC total score	3.04	(2.31)	0–10	3.28	(2.41)	2.92	(2.26)	1.33	0.185
DES-II	16.70	(13.07)	0–65.19	16.64	(12.15)	16.74	(13.55)	−0.06	0.952
PID-5-BF total score	21.89	(10.56)	1–58	22.23	(10.74)	21.72	(10.49)	0.42	0.676
PID-5-BF negative affectivity	6.87	(3.30)	0–15	5.86	(3.32)	7.40	(3.17)	−4.06	< 0.001
PID-5-BF detachment	3.94	(2.84)	0–13	4.15	(2.80)	3.83	(2.86)	0.97	0.331
PID-5-BF antagonism	2.87	(2.76)	0–14	3.59	(3.22)	2.49	(2.40)	3.44	0.001
PID-5-BF disinhibition	4.39	(2.88)	0–15	4.66	(2.77)	4.24	(2.93)	1.23	0.218
PID-5-BF psychoticism	3.83	(2.98)	0–13	3.97	(2.62)	3.75	(3.15)	0.63	0.531

*TEC, Traumatic Experiences Checklist; DES-II, Dissociative Experiences Scale-II; PID-5-BF, The Personality Inventory for DSM-5- Brief Form*.

We also examined the prevalence of traumatic experiences reported in the sample. Only 32 participants (9.9%) did not report any exposure to traumatic experiences in their life. However, the number of traumatic experiences among participants in this sample was low, with most participants reporting 1 (*n* = 65, 20.2%), 2 (*n* = 66, 20.5%), or 3 (*n* = 45, 14.0%) traumata. The most frequent traumatic experiences occurring in the sample were parentification (i.e., looking after parents and/or brothers and sisters during childhood; *n* = 108, 33.5%) and intense pain (e.g., due to an injury or a surgery, *n* = 106, 32.9%), the less frequent were war experiences (*n* = 1, 0.3%) and sexual harassment/abuse by family members (*n* = 1, 0.3%). Considering the experiences of abuse, 99 participants (30.7%) reported emotional abuse, 40 participants (12.4%) reported physical abuse, and 20 participants (6.2%) reported sexual abuse. Data concerning the age at which the first trauma was experienced was available for 274 participants (because responses such as “during my entire childhood,” “during adolescence,” “it started some years ago,” “as long as I can remember,” “I cannot exactly remember how old I was,” and the like, were excluded from this analysis). Among these participants, mean age of first trauma was 14.17 years (*SD* = 10.87), ranging from 0 (a life-saving surgery immediately after birth) to 55 years old.

Subsequently, the patterns of associations among TEC scores, DES-II scores, and PID-5-BF total and domain scores were examined. The results of this analysis are presented in Table 2. TEC scores were modestly but significantly and positively associated with DES-II and PID-5-BF scores, whereas the positive correlations between DES-II and PID-5-BF were stronger.

**Table 2:** Pearson's *r* correlations between traumatic experiences, dissociative experiences and maladaptive personality domains.

	**2**.	**3**.	**4**.	**5**.	**6**.	**7**.	**8**.
1. TEC total score	0.14*	0.22**	0.08	0.12*	0.23**	0.17**	0.20**
2. DES-II	–	0.50**	0.30**	0.21**	0.33**	0.40**	0.56**
3. PID-5-BF total score		–	0.69**	0.68**	0.72**	0.66**	0.68**
4. PID-5-BF negative affectivity			–	0.38**	0.34**	0.24**	0.44**
5. PID-5-BF detachment				–	0.37**	0.27**	0.44**
6. PID-5-BF antagonism					–	0.38**	0.54**
7. PID-5-BF disinhibition						–	0.51**
8. PID-5-BF psychoticism							–

TEC, Traumatic Experiences Checklist; DES-II, Dissociative Experiences Scale-II; PID-5-BF, The Personality Inventory for DSM-5- Brief Form;

* p < 0.05;

** *p < 0.01*.

**Figure 1:**
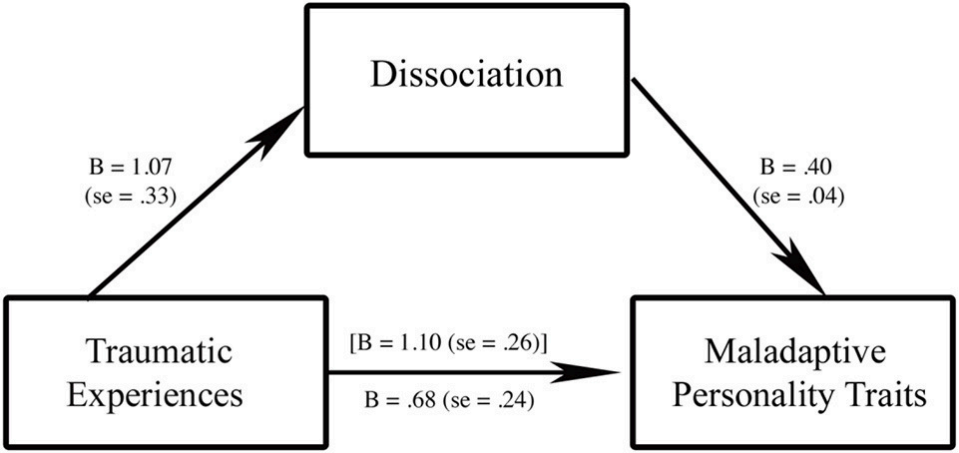
The mediating role of dissociation in the relationship between traumatic experiences and maladaptive personality traits. Traumatic experiences, Traumatic Experiences Checklist total score; Dissociation, Dissociative Experiences Scale–II total score; Maladaptive Personality Traits, The Personality Inventory for DSM-5-Brief Form total score; values in bracket square indicate the relationship between traumatic experiences (predictor) and maladaptive personality traits (outcome) without the inclusion of dissociation (mediator); all *p* < 0.01.

A mediation analysis controlling for gender, age, years of education, and marital status of participants showed that DES-II scores acted as partial mediators in the relationship between TEC scores (predictor) and PID-5-BF total scores (outcome). The hypothesized predictor (TEC total scores) was positively and significantly linked with both the proposed mediator (*B* = 1.07, *se* = 0.33) and the outcome (*B* = 1.10, *se* = 0.26). DES-II scores (mediator) were also positively related (*B* = 0.40, *se* = 0.04) with PID-5-BF total scores (outcome). The inclusion of DES-II scores in the model predicting PID-5-BF total scores by TEC total scores lowered the impact of TEC scores on PID-5-BF total scores (*B* = 0.68, *se* = 0.24), which, however, remained significant. The explained variance increased from 7.3%, *F*_(5, 316)_ = 4.44, *p* = 0.001, to 29.3%, *F*_(6, 315)_ = 31.33, *p* < 0.001 with the inclusion of dissociation in the model. Bootstrap analyses showed that the indirect effect of TEC scores on PID-5-BF total scores via DES-II scores was significant (C.I. 95%: 0.19–0.72), which was also confirmed by Sobel's test (*z* = 3.03, *p* = 0.002). Results from this mediation analysis are summarized in Figure 1.

Further mediation analyses were performed to examine the potentially mediating role of DES-II scores in the relationship between TEC scores and PID-5-BF domain scores. Table 3 summarizes the findings from these analyses. As shown in Table 3, DES-II scores acted as partial mediators in the relationship between TEC scores and PID-5-BF domain scores concerning antagonism, disinhibition, and psychoticism, and as total mediators in the relationship between tec scores and pid-5-bf domain scores concerning negative affectivity and detachment.

**Table 3:** Mediation models of the relationships between traumatic experiences (predictor) and maladaptive personality domains (outcomes) via dissociation (mediator).

	***a***	***b***	***c***	***c′***	***c – c′***		
**Outcomes**	***B*** **(C.I.95%)**	***B*** **(C.I.95%)**	***B*** **(C.I.95%)**	***B*** **(C.I.95%)**	***B*** **(C.I.95%)**	***R***^2^ **change**	**Total model** ***R***^2^
PID-5-BF total score	1.07 (0.42–1.72)	0.40 (0.32–0.49)	1.10 (0.59–1.62)	0.68 (0.21– 1.14)	0.43 (0.19–0.72)	0.22	0.29
Negative affectivity	1.07 (0.42–1.72)	0.07 (0.04–0.10)	0.18 (0.01–0.34)	0.10 (−0.05–0.26)	0.07 (0.03–0.14)	0.07	0.15
Detachment	1.07 (0.42–1.72)	0.05 (0.03–0.08)	0.15 (0.00–0.30)	0.09 (−0.05–0.24)	0.06 (0.02–0.11)	0.05	0.10
Antagonism	1.07 (0.42–1.72)	0.07 (0.05–0.10)	0.25 (0.11–0.40)	0.18 (0.03–0.32)	0.08 (0.03–0.14)	0.10	0.19
Disinhibition	1.07 (0.42–1.72)	0.08 (0.06–0.10)	0.24 (0.10 −0.38)	0.15 (0.02–0.28)	0.09 (0.04–0.15)	0.12	0.20
Psychoticism	1.07 (0.42–1.72)	0.13 (0.10–0.15)	0.29 (0.15–0.43)	0.15 (0.03–0.28)	0.13 (0.06–0.22)	0.27	0.34

*Predictor, Traumatic Experiences Checklist total score; Mediator, Dissociative Experience Scale–II; Outcomes, composite scores on the Personality Inventory for DSM-5- Brief Form; a, predictor on mediator; b, mediator on outcome; c, predictor on outcome without mediator; c′, predictor on outcome with mediator included in the model; c-c′, indirect effect; R^2^ change, increase in R^2^ after the inclusion of mediator in the model; Total Model R^2^, model including predictor, mediator, and covariates (gender, age, years of education, marital status). All total models were significant at p < 0.05*.

**Figure 2:**
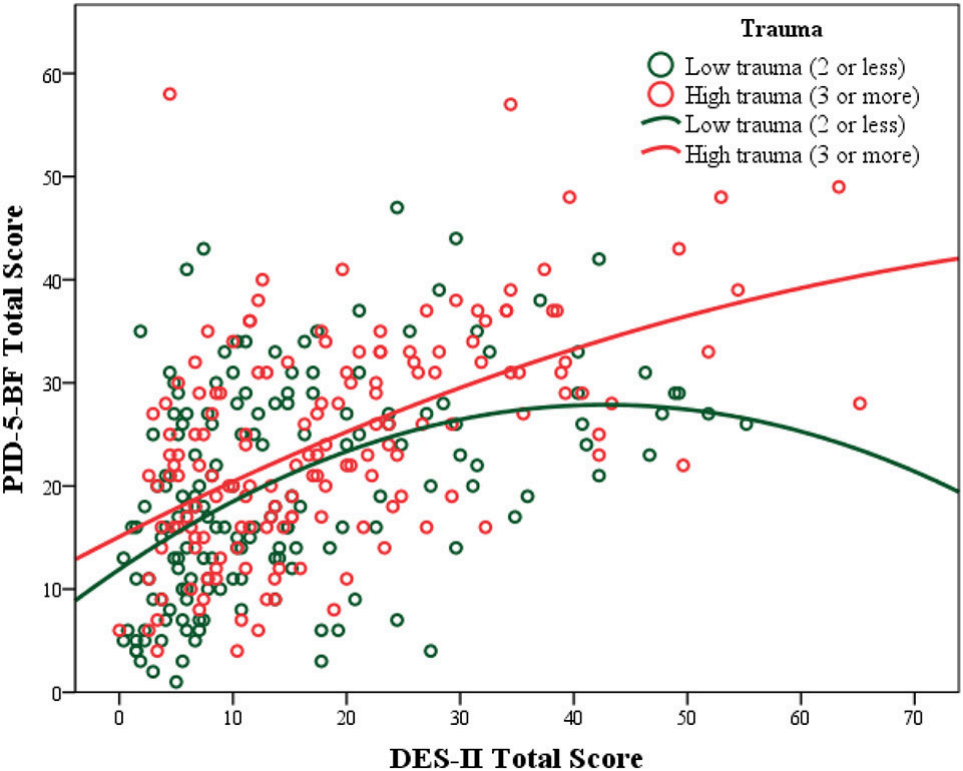
Predictive associations between dissociation scores and maladaptive personality scores at low and high levels of traumatization.

Finally, because linear regression analyses showed that DES-II scores predicted PID-5-BF total scores, *F*_(1, 320)_ = 107.35, *p* < 0.001, *R*^2^ = 0.251, the group of participants was split at the 50th percentile (median) of the TEC score distribution. This in order to identify participants in the sample who displayed low (TEC scores ≤ 2) and high (TEC scores > 2) levels of traumatization in this sample, and to examine whether the magnitude of the predictive association between DES-II scores and PID-5-BF total scores was related to different levels of traumatization. Different regression curves (linear, quadratic, cubic, exponential, and logistic) were tested, and it was verified that the relationship between dissociation and maladaptive personality features followed two distinct paths for people with low and high levels of trauma, with the best fitting curve for the data being a quadratic curve. In detail, the quadratic model was significant in the entire sample and in the two subgroups, *F*_(2, 319)_ = 56.36, *p* < 0.001, *R*^2^ = 0.261, and it explained 1% of additional variance with respect to the linear model. This analysis also showed that the predictive association between DES-II scores and PID-5-BF scores was stronger in the subgroup with high levels of traumatization (*R*^2^ = 0.292) than in the subgroup with low levels of traumatization (*R*^2^ = 0.209), and that the two regression curves followed different pathways (see Figure 2). The curve of those with high levels of traumatization was almost a straight line with no visible inflection while the parabola of the curve of those with low levels of traumatization showed a clear deflection at DES-II scores above 45.

## Discussion

The present study explored the relationships among psychological trauma, dissociation, and maladaptive personality domains. Consistent with previous studies on other nonclinical samples from the same country, our sample reported only low to moderate levels of psychological trauma (36), maladaptive personality traits (43, 44), and dissociative symptoms (3). Also consistent with the literature, males reported higher antagonistic features than females, and females reported higher negative affectivity than males (44). The main aim of our study was to test a conceptual model in which the predictive association between trauma and maladaptive personality functioning would be mediated by dissociation. The results of the mediation analyses supported this model.

In line with previous empirical findings and clinical considerations on the close relationship between complex trauma and dissociative experiences (45–47), we observed that increased numbers of traumatic experiences were associated with increased levels of dissociation in our study. Furthermore, we observed that traumatic experiences had both direct and indirect effects on global maladaptive personality functioning. Traumatic experiences significantly and positively predicted all maladaptive personality domains after controlling for sociodemographic variables (gender, age, years of education, and marital status), and they continued to significantly predict the domains of disinhibition, psychoticism, and antagonism after the inclusion of dissociation in the mediation models. Therefore, our results are generally consistent with previous studies showing that traumatic experiences can have a significant impact on the development of specific maladaptive personality domains, and especially those domains linked with self-regulation, identity integration, and difficulties with relatedness.

We also found that dissociative experiences always acted as significant mediators in the relationships between levels of traumatization and maladaptive personality domains scores. This result mirrors findings from a previous study on the relationships among trauma, dissociation, and psychopathology (5), and supports a wealth of clinical literature (1, 7–10, 14, 48, 49) suggesting that the development of maladaptive personality traits may result from complex traumatization (50–55) via an excessive activation of the dissociative processes (11, 16).

However, dissociation acted as a partial mediator, not as a full mediator, in the relationship between trauma score and overall maladaptive personality scores. A possible explanation of this finding is rooted in the fact that not all distressing experiences are processed through dissociation. Many traumatic experiences occur in fact in normal consciousness and are retained in accessible and retrievable memory (56–58). Thus, consistent with recent theory conceiving psychological trauma as a complex factor in which specific traumatic experiences may have different probability to combine and may relate differently to dissociative processes and psychopathology (5), it could be possible that several types of distressing experiences are tolerable to an individual's mind—perhaps because they occurred just once in life and/or at an adult age (when the personality and the capacity for affect regulation and mentalization are already developed) and/or when effective social support was available to buffer their effects. Such experiences may be encoded and processed at a conscious level, although they also may continue to constitute a source of distress for the individual. Other distressing experiences, which perhaps occurred more frequently, and/or started at an early age generating a maladaptive developmental cascade, and/or were extremely disorganizing, could be instead coded in altered and dissociative states of consciousness, with a stronger influence on the development of maladaptive personality functioning (4, 7, 13, 44, 59).

In this respect, dissociation may represent a mental process that can be used in adaptive or maladaptive ways in response to distressing experiences occurring in an individual's life. In fact, regression curve analyses showed two different pathways for the relationship between dissociation and maladaptive personality functioning, in which the numbers of trauma played a pivotal role. For individuals exposed to low numbers of traumatic experiences, personality functioning was only barely linked to dissociation, and actually the highest scores on dissociation corresponded to a decrease in maladaptive personality scores, suggesting that dissociation in this situation can act as a normal and even positive function of the mind, likely related to normal self-absorption phenomena and creative responses to distressing events (60). Thus, we could hypothesize that when the mind is exposed to low numbers of distressing experiences, dissociation can be used flexibly as a defense mechanism that protects the self from being overwhelmed by distressing stimuli or by excessive anxiety. On the contrary, for highly traumatized individuals, dissociation may become pervasive, jeopardizing their entire mental life (3). In these circumstances, the mind seems to keep the score (61) of previous traumata, and dissociation may become a psychological organizer of the entire self (11, 13), playing a key role in the development and consolidation of maladaptive personality domains.

Cumulatively, these considerations may suggest that dissociation as a psychological process maintains its own specific role in personality functioning, with different levels of trauma-related dissociation (or, better to say, with different levels of activation of the dissociative process following different levels of traumatization) linked to different levels of impairments in personality functioning.

In conclusion, our study supports the general hypothesis that maladaptive personality functioning is linked with trauma-related dissociation. However, as with all research, the present study comes with a number of limitations. First, the sample is constituted only by adult volunteers from an Italian isle, which limits the generalizability of our results. Second, causal interpretations of the relationships among traumatic experiences, dissociation, and personality are invoked based on theory, but the findings should be cautiously interpreted, and it is impossible to directly extend them to other samples due to the cross-sectional design of the study and the nonclinical nature of the sample. In this respect, longitudinal studies with clinical and nonclinical samples are warranted. Third, we did not distinguish between different types of trauma and the age at which they occurred, and actually we weighed all traumatic experiences equally, while research and clinical experiences suggest that different types of trauma might differently affect the development of personality. This approach to research data was clearly useful to empirically test the theoretical model on complex traumatization (5, 10) already illustrated in the present work and in other articles with respect to personality functioning, but we are aware that such approach to hypothesis-testing was taken at the price of potentially obscuring the relationships between specific characteristics of traumatic experiences and specific domains of maladaptive personality.

## Conclusions

Even considering its limitations, this study shed some new light on how trauma and dissociation may undermine an individual's personality functioning. From a psychodynamic perspective, clinical work with traumatized patients is aimed at reestablishing the unity and uniqueness of self and personal identity by fostering mentalization, affect regulation, and a basic sense of safety and trust toward interpersonal relationships (4, 10–12, 14, 16, 62–69). So, understanding whether the excessive activation of the dissociative process has been so pervasive for an individual to deviate his or her entire organization of personality is a first step in uncovering the impact (and sometimes even the presence, in some cases of dissociative functioning with severe amnesia) of psychological trauma. This kind of knowledge can certainly help clinicians choose the best treatment available for people who display maladaptive personality traits.

## Ethics statement

This study was carried out in accordance with the recommendations of the American Psychological Association (APA) and the Italian Association of Psychology (AIP). The protocol was approved by the Internal Review Board for Psychological Research (IRB) of the UKE-Kore University of Enna (10th October 2016). All subjects were given a complete description of the study and provided written informed consent in accordance with the Declaration of Helsinki.

## Author contributions

AS designed the study and managed the research process. FG and AC collected and analyzed the data. AG, FG, and AC performed the literature review. AG, FG, and AS took primary responsibility for initial drafting. VC contributed with relevant theoretical and clinical inputs. AG and AS were responsible for subsequent collation of inputs and redrafting. All authors critically revised the manuscript and approved its final version.

### Conflict of interest statement

The authors declare that the research was conducted in the absence of any commercial or financial relationships that could be construed as a potential conflict of interest.
